# An Optical Modeling Framework for Coronavirus Detection Using Graphene-Based Nanosensor

**DOI:** 10.3390/nano12162868

**Published:** 2022-08-20

**Authors:** Amir Maghoul, Ingve Simonsen, Ali Rostami, Peyman Mirtaheri

**Affiliations:** 1 Optical/FNIR Laboratory of Biomedical Group, Department of Mechanical, Electronics and Chemical Engineering, OsloMet–Oslo Metropolitan University, 0167 Oslo, Norway; 2 Department of Physics, NTNU—Norwegian University of Science and Technology, 7491 Trondheim, Norway; 3 Photonics and Nanocrystals Research Laboratory (PNRL), Faculty of Electrical and Computer Engineering, University of Tabriz, Tabriz 5166614761, Iran

**Keywords:** COVID-19 particle model, COVID-19 spikes, reflectance, graphene-based nanosensor, gold nanodisks, blood sample

## Abstract

The outbreak of the COVID-19 virus has faced the world with a new and dangerous challenge due to its contagious nature. Hence, developing sensory technologies to detect the coronavirus rapidly can provide a favorable condition for pandemic control of dangerous diseases. In between, because of the nanoscale size of this virus, there is a need for a good understanding of its optical behavior, which can give an extraordinary insight into the more efficient design of sensory devices. For the first time, this paper presents an optical modeling framework for a COVID-19 particle in the blood and extracts its optical characteristics based on numerical computations. To this end, a theoretical foundation of a COVID-19 particle is proposed based on the most recent experimental results available in the literature to simulate the optical behavior of the coronavirus under varying physical conditions. In order to obtain the optical properties of the COVID-19 model, the light reflectance by the structure is then simulated for different geometrical sizes, including the diameter of the COVID-19 particle and the size of the spikes surrounding it. It is found that the reflectance spectra are very sensitive to geometric changes of the coronavirus. Furthermore, the density of COVID-19 particles is investigated when the light is incident on different sides of the sample. Following this, we propose a nanosensor based on graphene, silicon, and gold nanodisks and demonstrate the functionality of the designed devices for detecting COVID-19 particles inside the blood samples. Indeed, the presented nanosensor design can be promoted as a practical procedure for creating nanoelectronic kits and wearable devices with considerable potential for fast virus detection.

## 1. Introduction

Since 2019, with the emergence of COVID-19, the world society has faced a human disaster in terms of the mortality rates caused by this disease. Due to the increasing outbreak of COVID-19 infectious disease, the World Health Organization (WHO) decided to classify it as a pandemic on 4 March 2020 [1]. In the meantime, because of the rapid transmission rate of COVID-19, the WHO has also established a guideline approach based on ASSURED [2] as a benchmark that needs to be followed. It includes affordable, sensitive, specific, user-friendly, rapid and robust, equipment-free, and deliverable to end-users to break the race of disease transmission with the most appropriate diagnostic tests for resource-constrained situations [3]. However, considering the complex nature of this virus and the outbreak of the new variants, it has been a terrible challenge for nations to control the person-to-person transmission procedure of the COVID-19 disease [4,5]. Hence, a precise identification under a reliable test seems vital in the first line of defense against corona infection. Currently, the standard techniques for the COVID-19 test have relied on two main categories: molecular tests and serological tests. In detail, molecular strategies primarily use polymerase chain reaction (PCR) as a real-time technique to reveal COVID-19 infection in individuals, which involves drawbacks such as the high cost of doing the test, it being time-consuming, and the need for laboratory processing to analyze the samples with more accuracy. Despite these challenges, the PCR test still counts as the most convenient method from a sensitivity point of view the countries have to use for COVID-19 infection tests in the available situation [6,7]. As such, in the serological test [8], the amount of IgG and IgM antibodies in the blood serum sample of the patient is an indication of corona infection and presents important surveillance data about the previous viral diseases, though it can not detect the active cases [9]. Based on the mentioned issues, fast and well-time detection devices are urgently needed to minimize the damage arising from the COVID-19 pandemic and increase the immunity level against coronavirus infection in people.

For the time being, most research efforts are focusing on ultra-fast coronavirus detection systems to isolate the affected persons and break the COVID-19 transmission chain. In between, using the nanosensors due to their unique and extraordinary properties in ultra-rapid detection of viral particles and their considerable sensitivity have attracted much attention [10,11]. Indeed, nanomaterials, especially 2D substances such as graphene [12] embedded into the nanosensors, can create a significant potential for efficient sensing and detection of different analytes and molecules on the nanoscale [13,14]. Recently, new developments in the field of graphene-based structures associated with quantum technology are emerging, which can be enhanced the sensing capability [15,16]. Along with this, using electrochemical techniques in the design of graphene sensors has also attracted much attention, providing high-precision sensory devices [17,18]. As such, the multimode surface plasmon resonance absorber design based on graphene material can be developed as a series of multitasking sensors with the potential for different-size particle detection [19].

**Figure 1:**
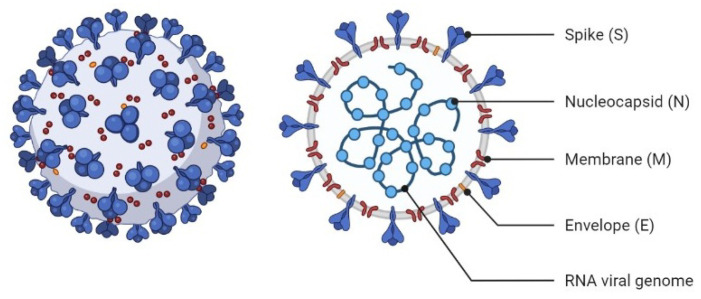
A schematic of the human coronavirus structure.

From a structural point of view, graphene consists of carbon atoms formed in a honeycomb lattice. Each carbon contributes to three intralayer $sp^{2}$ or sigma (σ) bonds with its three neighboring carbon atoms. These bonds, named the covalent band, can provide an extreme potential for the graphene layer to sense. Interestingly, this strength in the bond can be changed by the presence of defects or attaching impurities, which is the main reason for using the graphene layer in sensory applications [20,21]. Further, graphene and its derivations, due to its large surface area, unique optical and magnetic properties, and high elasticity, which can apply to microorganism detection, have become the interest of many scientists [22]. In this context, a correct understanding of the physical characteristic of coronavirus in the optical range corresponding to the size of a COVID-19 particle can also outline very new knowledge to support the design approaches of the modern nanosensors for ultrafast detection [23,24]. For this, mathematical modeling as a powerful tool provides an effective potential for analyzing and characterizing viral infections epidemiologically and can create better insights into the viruses’ dynamics [25]. Figure 1 shows a graphical diagram of the human coronavirus structure associated with the genomic sequence.

This paper presents an optical simulation framework for coronavirus detection through a nanosensor configured by the graphene layer. At the start, an engineering model of the COVID-19 particle based on the established theoretical concepts is extracted, and then, for the first time, the optical behavior of the coronavirus is simulated and analyzed over the optical range. In detail, the reflectance parameter is achieved under the interaction between light and the COVID-19 particle model. In addition, the impact of physical parameters changes, such as the size of the COVID-19 particle and the dimension variation of spikes around the COVID-19 particle on the optical characteristic of the COVID-19 model, are explored. The concentration effect of COVID-19 particles upon the reflectance parameter is also calculated, and its simulation results are evaluated. With this in mind, we propose an exciting framework for the development of a nanochip based on the embedded graphene material for COVID-19 particle detection in the blood serum sample. The suggested kit is designed based on the fundamental concepts governing plasmonic modes and the effective refractive index in physics, which is discussed extensively.

## 2. Model System

**Figure 2:**
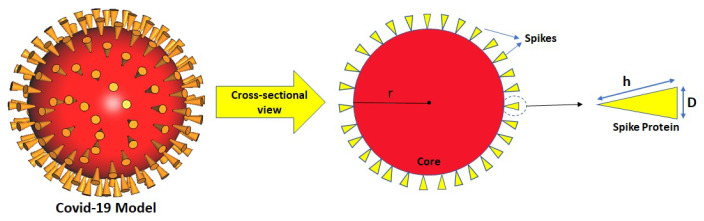
An engineering model of COVID-19 particle associated with the surrounding spikes (N=147).

Coronaviruses are an extensive family of enveloped viruses with the feature of positive-sense RNA that basically infects animals as a host environment and transmits to the human body. Amongst them, three kinds of such viruses, including SARS-CoV, MERS-CoV, and SARS-CoV-2, can create respiratory problems at different levels, from a mild cold to killer infection [26,27]. From a morphological point of view, there is a certain number of spike proteins on the coronavirus surface, and these spikes facilitate the process of entering the host cell. Hence, the physical behavior characterization of spikes, known as a dangerous factor, can provide helpful knowledge to understand better the coronavirus’s operational manner [24,28]. In addition, focusing on the morphology of COVID-19 particles based on the available literature [29] and the size of these viruses, which is approximated between 60 and 140 nm in diameter [30], can be created an increasing motivation to investigate the physical characteristic of the coronavirus in the optical range. Because most viral particles of the corona’s family are spherical [31,32], in this paper, a spherical shape is assumed as the central core of the COVID-19 model to which conical shapes inverted like spikes are attached, as shown in Figure 2. For the primary structure of the COVID-19 model assumed in this work, the diameter of the COVID-19 model (2r) and the length of spikes (*h*) are assumed to be 140 nm and 20 nm, respectively (numbers extracted from Ref. [24]). In addition, the base of the cone is 8 nm in diameter (*D*), and the number of spikes around the core of the COVID-19 model is assumed to be N=147. In detail, the data allocated to the dimensions of protein spikes and the modeled COVID-19 structure are concluded from the structural basics of virology [33]. All simulation results presented in this paper are carried out by the use of numerical methods based on the finite integration technique (FIT) implemented in CST Microwave Studio.

## 3. Simulation Results

### 3.1. Optical Modeling and Characterization of COVID-19 Particle

In order to achieve a good perception of the operational dynamics of the coronavirus based on mathematical modeling, we need to consider some essential parameters to implement a realistic model of the COVID-19 particle in the simulation environment of the CST Microwave Studio. For this reason, we first assume the background medium in which the COVID-19 particle is modeled to be water because it constitutes the principal part (95 percent) of blood plasma [34,35]. It is worth mentioning that human blood consists of erythrocytes (red blood cells), leukocytes (white blood cells), and thrombocytes (platelets). Of these cells, the red blood cells are the most abundant, about 45 percent, and they are also the largest, about 7 microns in diameter. Therefore, these cells play a key role in determining the propagation of light through blood at the microscopic level. Moreover, the blood cells are floating inside the blood’s plasma, which primarily consists of water [36]. In the following, the engineering model of the COVID-19 particle based on the dimensions given in the previous section is implemented. From the physics perspective at the nanoscale, the optical properties of particles can be defined by the refractive index of the ingredient of the substance. Inspired by this, we approximate the refractive index of spikes in the presented COVID-19 model equivalent to fatty tissue [35] since a significant part of the spike proteins surrounding the COVID-19 particle is comprised of lipid [37]. Additionally, according to the available data related to the refractive index of the COVID-19 particle reported in Ref. [38], the refractive index of the COVID-19 model is considered to be “−0.967251”. To extract the optical characteristic of the proposed COVID-19 model, the reflectance is calculated in the following simulations. Reflectance is related to the ratio of the wave intensity reflected from the COVID-19 particle ($I_{r}$) to the incident wave intensity impinging on the same particle ($I_{0}$), and it is defined by [39]:

(1) $$R\;\left(dB\right)=10\;\log_{10}\left(\frac{I_{r}}{I_{0}}\right).$$

**Figure 3:**
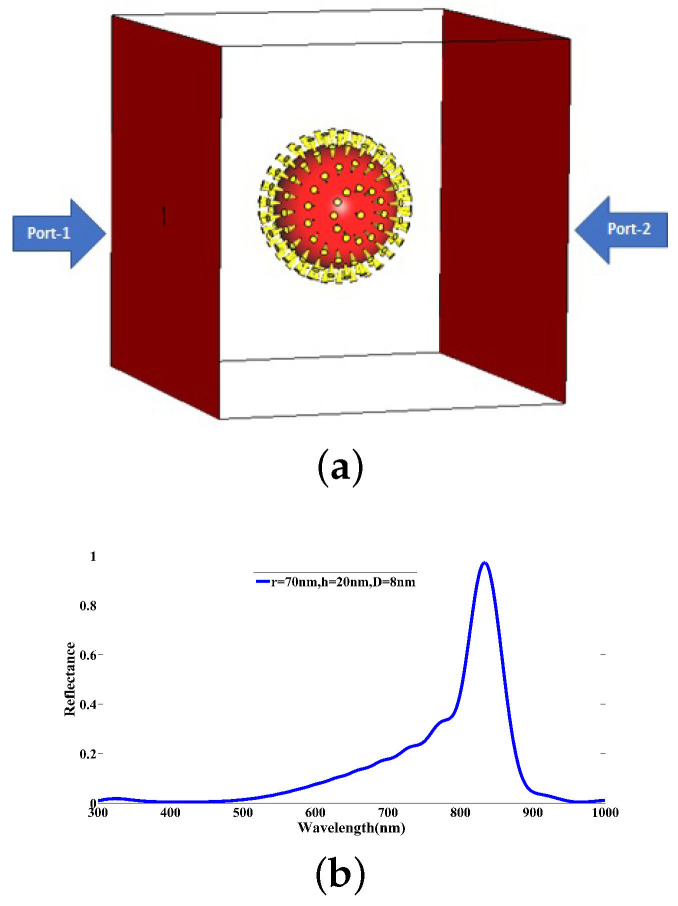
(**a**) Stimulation schematic of COVID-19 particle model defined by r=70 nm, h=20 nm, D=8 nm, N=147, and implemented in CST with input and output ports. (**b**) The simulated reflectance spectrum of the COVID-19 particle model under the light incidence.

For stimulation of the COVID-19 particle model, two wave ports are located at the two sides of the implemented structure as shown in Figure 3a, one as an input port for applying the excitation signal and the other as an output port. A Gaussian signal with the electric field intensity of 1 V/m is used for the excitation signal. In order to satisfy the boundary conditions regarding the presented structure, we utilize the option *open and space* to analyze the COVID-19 model. Owing to the importance of optical characterization of the COVID-19 structure earlier, we choose an optical wavelength band ranging from 300 nm to 1000 nm for all simulations, covering the visible-near infrared (NIR) bands. Interestingly, the simulated reflectance arising from the initial COVID-19 model in Figure 3b indicates a significant peak at the wavelength 833.92 nm over the spectral response.

**Figure 4:**
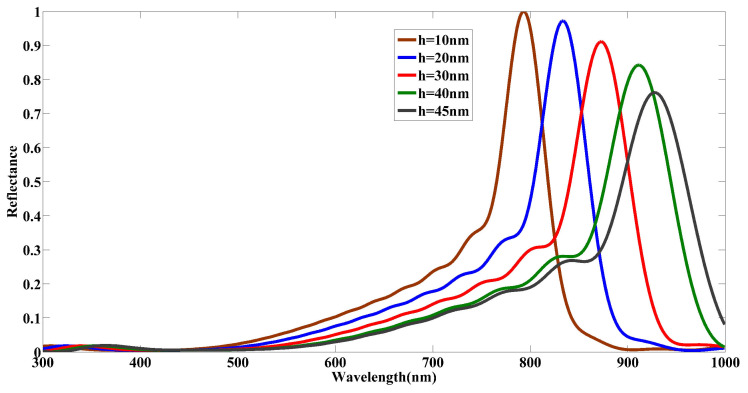
Numerically calculated reflectance spectra for a single COVID-19 particle for five spike lengths *h* in the range from 10 to 45 nm. The values of the remaining geometric parameters defining the COVID-19 particle were r=70 nm, D=8 nm, and N=147.

To evaluate the impact of the structural variations of the COVID-19 particle model, we first change the considered initial length of the spikes (h=20 nm) to h=10 nm, 30 nm, 40 nm, and 45 nm while keeping the rest of the geometrical parameters of the primary COVID-19 model fixed. The simulation results presented in Figure 4 are expressed as a variation of the electromagnetic scattering profile (the form of the spectra) and, in particular, in the wavelength of the resonance (the maxima of the reflectance spectra). In detail, the effective refractive index of the COVID-19 model resulting from the electromagnetic modes generated changes under the manipulating of the spike’s length. Consequently, for increasing length of the spikes *h*, a redshift and monotonous decreasing reflectance at the wavelength of the resonance can be observed in the simulated spectra (Figure 4). Table 1 presents the quantitative data of the calculated reflectance and its corresponding wavelength at the reflection peak. As well highlighted, a downward trend in reflectance intensity associated with a wavelength shift from 793.73 nm to 927.86 nm happens.

**Figure 5:**
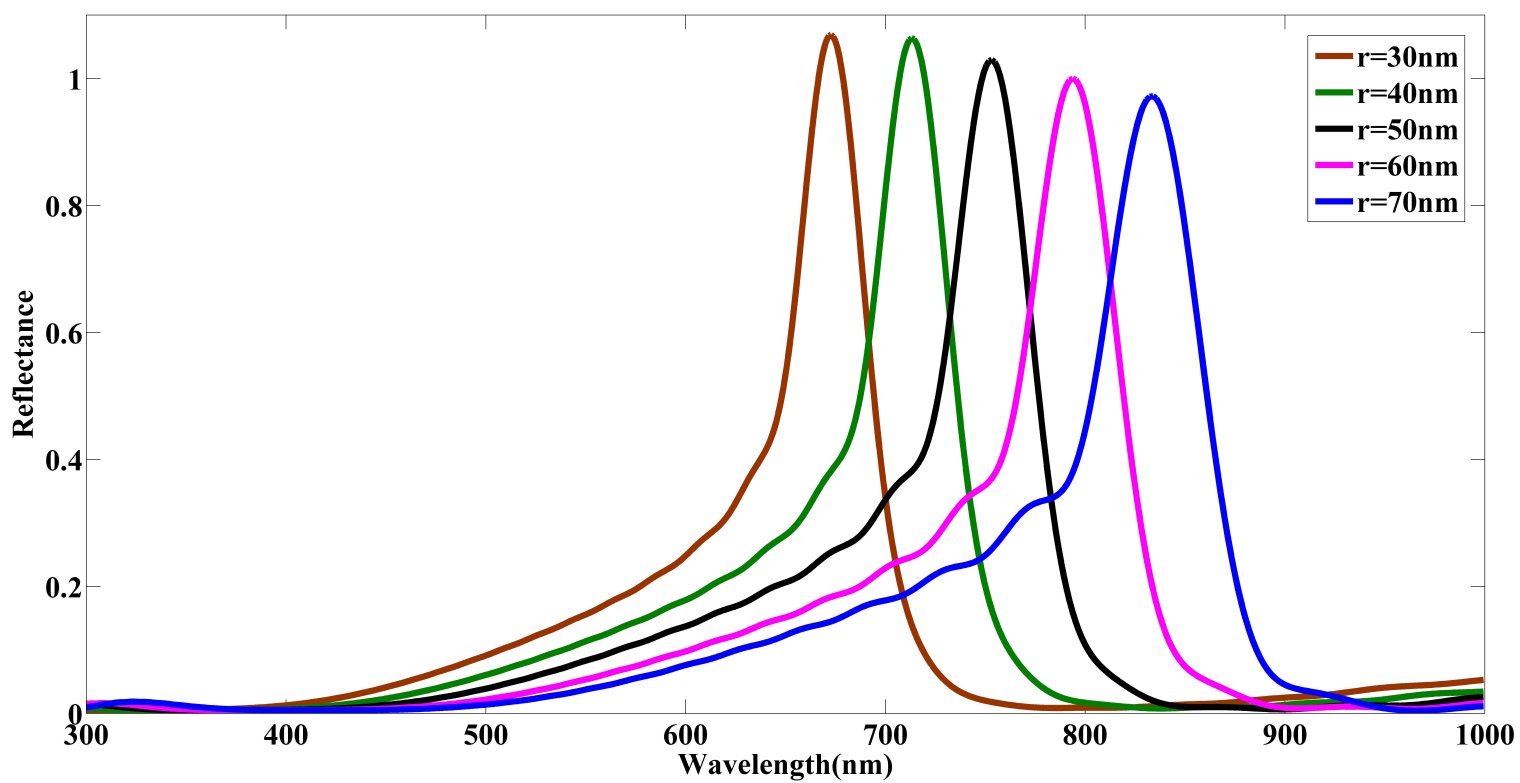
The simulated reflectance spectra for COVID-19 particles of varying core radius *r*. The values of the remaining geometric parameters defining the COVID-19 particle were h=20 nm, D=8 nm, and N=147.

Next, the radius *r* of the COVID-19 particle’s core is varied while the length of the spikes is kept constant at h=20 nm and D=8 nm. The simulation results in Figure 5 demonstrate that the size of the COVID-19 particle plays a key role in the reflectance parameter and the wavelength in which the reflection peak occurs. There is a blueshift with a gradual increase in the maximum value of reflectance on the achieved spectral responses over the reflection band when the radius of the COVID-19 model is reduced. In fact, reducing the size of the COVID-19 particle alters the scattering profile of the reflected electromagnetic waves, resulting in a change in the effective refractive index; thus, blueshift occurs in the reflection band. In addition, it can be seen in Figure 5 that the reflectance’s peak is more than 1 by shrinking the radius of the COVID-19 model particle. With attention to the relationship of the reflectance formula in classical physics [40] and considering the negative refractive index of the core of the COVID-19 model in the presented structure, the reflectance’s peak can be over 1. A quantitative assessment of the variations of the optical characteristic of the COVID-19 model under the manipulation of the particle’s radius is shown in Table 2 in which a wavelength shift from 672.78 nm to 833.92 nm is associated with a falling trend in the intensity is seen. As a result, the structural changes of the COVID-19 particles can significantly influence the optical spectroscopic response of the samples.

**Figure 6:**
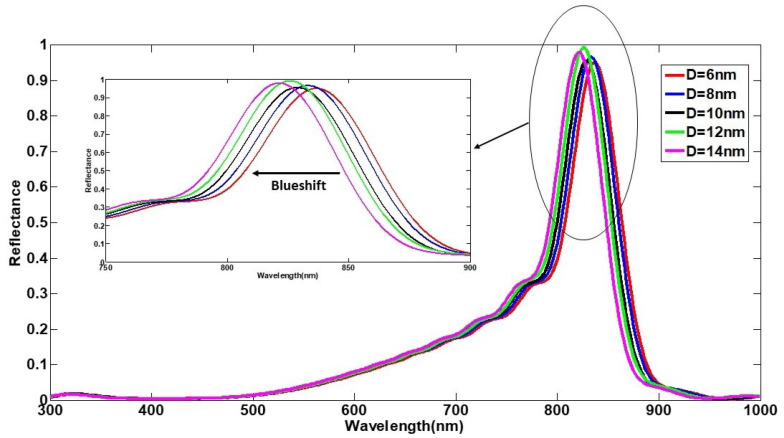
The simulated reflectance spectra of the COVID-19 particle model under changing the base diameter of COVID’s spikes. The values of the remaining geometric parameters defining the COVID-19 particle were r=70 nm, h=20 nm, and N=147.

In order to acquire more knowledge of the manipulation of spikes’ size, the base diameter of the conical spike available in the model is changed, ranging from 6 nm to 14 nm, while the rest of the dimensions are constant and similar to the initial COVID-19 model. The simulation results indicate that a blueshift happens in the achieved spectra by changing the base diameter of spikes on the reflection band, as demonstrated in Figure 6. To gain a better understanding, a quantitative evaluation regarding the variation of the reflectance parameter is presented in Table 3 and a wavelength displacement is well observed from 837.18 nm to 821.12 nm. Indeed, the change in the size of spikes manipulates the components of the electromagnetic field in the reflected wave, which leads to varying the scattering profile, which appears like a blueshift upon the reflection band under the interaction of the electromagnetic fields. Since the spectroscopic impact of the number of spike proteins surrounding the COVID-19 particles was recently studied extensively [24], we have chosen not to repeat such a study. Instead, we have assumed the number of spikes proteins (for each virus) to be fixed at N=147 throughout this study.

**Figure 7:**
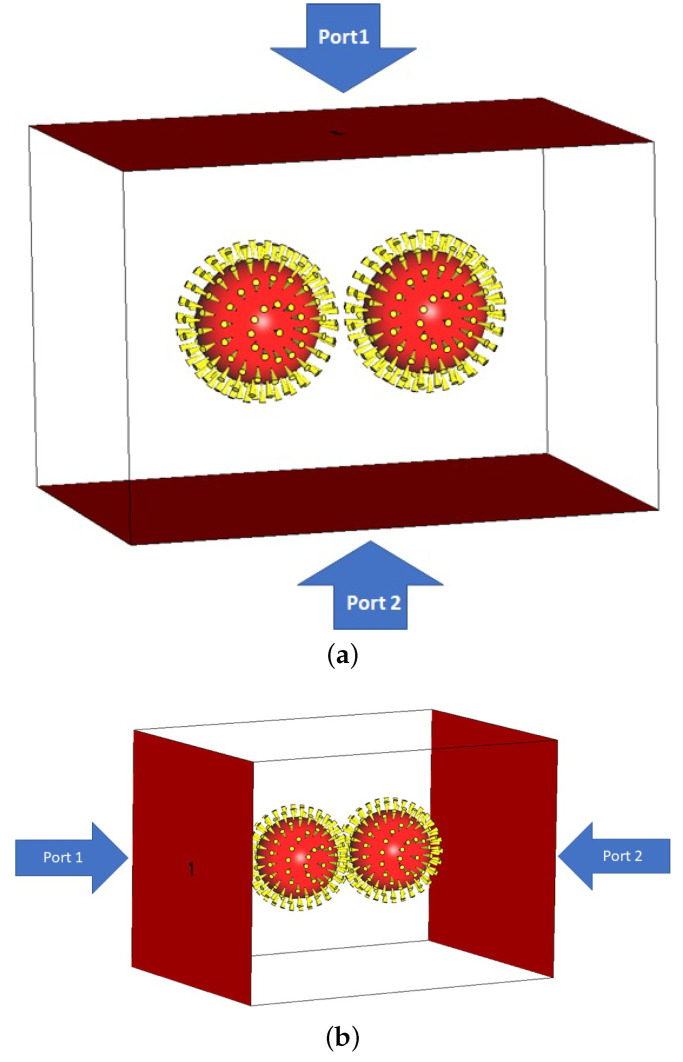
Stimulation schematic diagram for the demonstration of the COVID-19 model density implemented in CST with input and output ports for two states of optical excitation from (**a**) the top and (**b**) the side. The geometric size of COVID-19 particles’ structures is defined by r=70 nm, h=20 nm, D=8 nm, and N=147.

Following this, we aim to understand the optical behavior of COVID-19 particles’ concentration and investigate the density effect of the coronavirus by analyzing the optical characteristic through numerical simulation. For this, two COVID-19 models are first implemented in the software environment, as seen in Figure 7. Focusing on the density effect of COVID-19 particles, it is also essential to pay attention to the direction of the incident light because adding another COVID-19 particle creates an asymmetrical status in the configuration, altering the scattering profile. Hence, we choose two structural models for our simulations to explore the impact of the incidence direction as well as the effect of particle concentration. The size of these COVID-19 particles is similar to the initial COVID-19 model, and the center-to-center distance of particles is assumed to be 179 nm at the start of the simulation and then changes to 199 nm and 219 nm to explore the distance effect of COVID-19 particles. To apply the excitation signal with the electric intensity of 1 V/m for COVID-19 particle stimulation, two wave ports, such as input and output according to Figure 7a,b, respectively, are considered.

**Figure 8:**
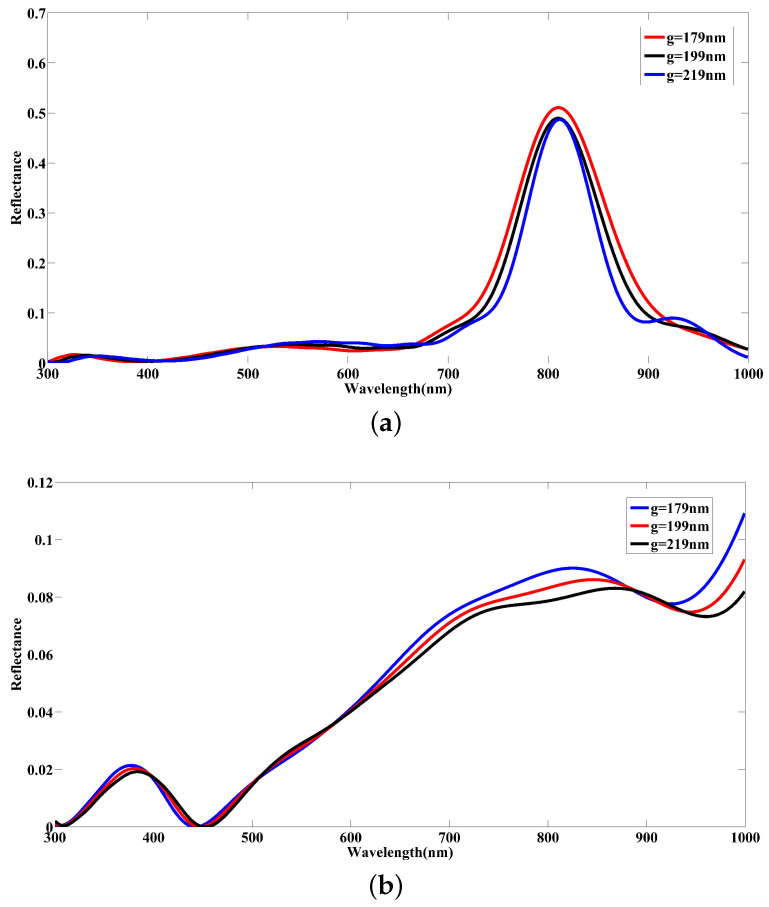
The simulated reflectance spectra arising from the demonstration of COVID-19 model density under changing the distance between COVID-19 particles for two states of the light incidence from (**a**) the top and (**b**) the side directions.

The simulation results presented in Figure 8 show a significant deformation in spectral responses for two kinds of stimulation. Interestingly, we can observe a tangible narrowing in reflectance spectra and a visible drop in reflection intensity under increasing the distance between two COVID-19 particles while the incident optical signal is applied from the top, as in Figure 7a. Additionally, the result of the reflected electromagnetic field components under the interaction between incident light and two COVID-19 particles indicates a destructive interference effect to reduce the reflection intensity and shape a narrowing result in the reflectance spectra, as shown in Figure 8a. With changing the direction of the excitation signal and light incidence from the side, as in Figure 7b, the reflectance is decreased dramatically due to the high absorbance rate of the medium, as seen in Figure 8b. As a result, the incidence angle of the optical stimulation signal plays a determining role in detecting COVID-19 particles during the spectroscopy of samples under test. Moreover, the density of COVID-19 particles can reduce the amount of reflectance because of destructive interference between electromagnetic field components reflected and the high absorption rate of the plasma medium modeled by water in which the COVID-19 particles are placed in this work.

### 3.2. Coronavirus Detection Using Graphene-Based Nanosensor

**Figure 9:**
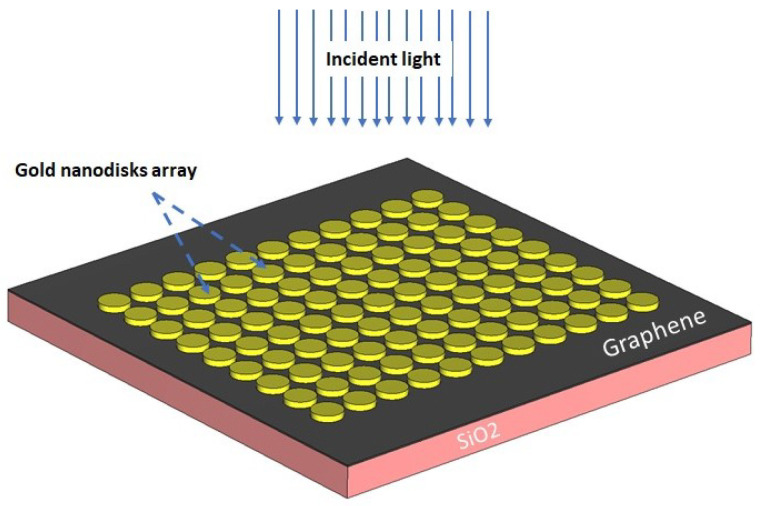
A schematic diagram of the proposed nanosensor associated with the optical stimulation signal.

As mentioned earlier, the rapid transmission nature of COVID-19 has created a difficult and challenging situation for all the ordinary activities of humans and causes excessive fear for people [41]. On this basis, rapid COVID-19 detection is known as an efficient approach that can play a vital role in preventing coronavirus outbreaks. The use of sensors, especially nanosensors, as a potent tool for early notification of symptoms of corona disease is a conventional strategy that is emerging to monitor and control the trend of this dreadful disease [42,43]. Among the nanomaterials used in the configuration of nanosensors, graphene has recently been of particular interest due to its unique optical properties [3,12,44]. Graphene is a two-dimensional nanomaterial containing carbon atoms arranged in a honeycomb lattice in terms of optical properties. Utilizing light incidence, the surface plasmon polaritons of graphene can also provide sharp, broad peaks in the optical range [45,46]. With this in mind, here we propose a design framework for a graphene photonic structure based on established theoretical concepts and numerical simulations in which graphene functions as an innovative material that has the potential for identifying a blood sample involving COVID-19 particles. In view of silicon oxide’s high biocompatibility and functionality, we select a substrate of SiO_2_ with an area of 1.6 µm × 1.6 µm and a thickness of 100 nm at the start of the design procedure. A set of complex data available in the CST’s library are used for the refractive index of silica (SiO_2_) [47,48]. A thin layer of graphene material with a thickness of 5 nm is placed on top of the silica substrate. To define the optical properties of graphene sheet in the CAD, the physical properties of graphene are considered as follows: the Fermi potential is regarded at 0.4 eV and the temperature and relaxation time is set to 300 K and 1 ps [49,50]. Next, several gold nanodisks are positioned on the graphene layer, as shown in Figure 9. The gold nanodisks are within the size of 100 nm in the base diameter and 20 nm in height. Further, a Drude model is considered to be determined the optical properties of gold material. In this model, the permittivity of bulk gold, the plasma frequency, and the damping constant are defined by $\epsilon_{\infty }=1$, $\omega_{p}=1.37\times 16\;s^{-1}$, and $\omega_{r}=1.23\times 14\;s^{-1}$, respectively [51,52].

**Figure 10:**
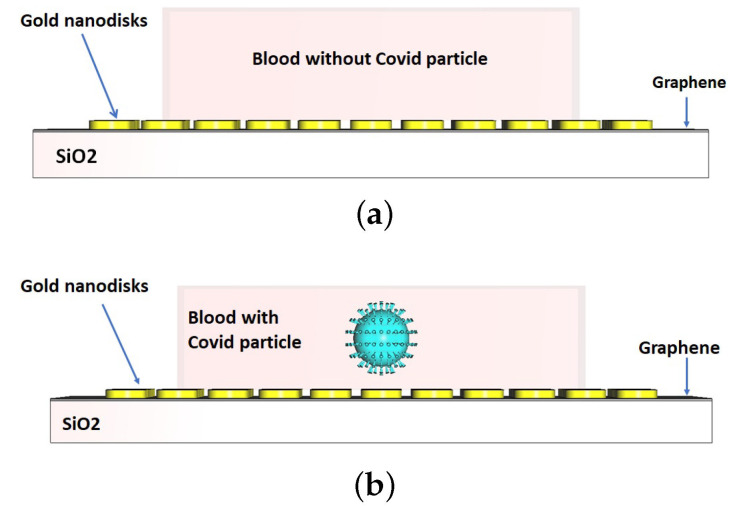
A graphical screen of the nanosensor under test with blood samples in two states: (**a**) without COVID-19 particle and (**b**) with COVID-19 particle. The geometric size of COVID-19 particle model is based on r=70 nm, h=20 nm, D=8 nm, and N=147.

Like before, a Gaussian pulse as incident light from the topside for excitation of the designed kit is utilized, illustrated by a wave port in the CST’s simulation environment. In order to evaluate the performance of the designed graphene kit, it is required to test a blood sample with and without COVID-19 particles. For this purpose, a cube-shaped blood sample with the dimensions 500 nm×500 nm×250 nm based on the established data is modeled and placed on the gold nanodisks, as demonstrated in Figure 10a,b. The refractive index of blood is approximated by the complex data related to hemoglobin in [35]. To clarify further, a blood sample is first placed on the nano kit, and the reflectance is calculated under the incidence of light in the desired range from 300 nm to 1000 nm. Then, a blood sample model associated with a COVID-19 particle is considered, while the structure of the COVID-19 particle is the same initial COVID-19 model that was mentioned in the previous section. Significantly, we choose the single COVID-19 particle inside the blood sample for the start of the simulation in this section in order to avoid the mutual coupling effect and interaction of the reflected electromagnetic fields arising from more particles and find a precise understanding of the functionality of the nano kit. To visualize the results better, we choose two wavelength windows (300– 600 nm and 600– 1000 nm) over the reflection band of the designed nanosensor.

**Figure 11:**
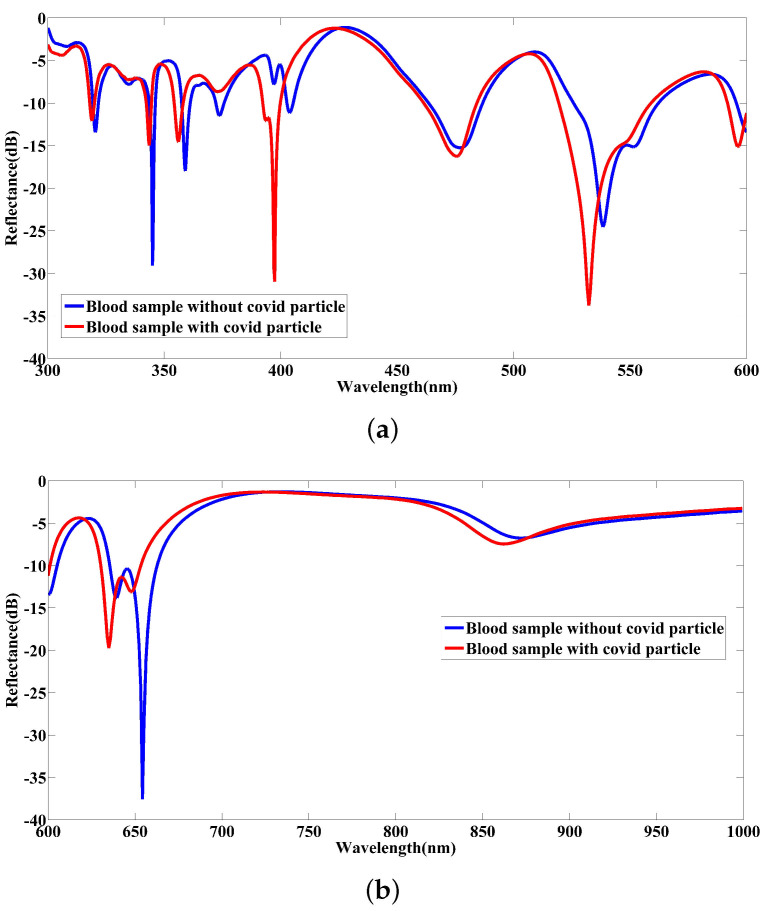
The simulated reflectance spectra arising from the proposed nanosensor when testing blood samples without COVID-19 particle and with a single COVID-19 particle for wavelengths interchanged between (**a**) 300−600 nm and (**b**) 600−1000 nm.

The simulated results in Figure 11 indicate a tangible displacement in spectral responses achieved in the reflection band when tested with healthy and infected blood samples. In fact, adding the COVID-19 particle changes the optical properties of healthy blood samples. In other words, the effective refractive index of a healthy blood sample alters with the presence of available COVID-19 particles. This causes the modal components of electromagnetic waves reflected to be changed. These variations result in a shift of reflectance spectrum in the considered range. It is worth mentioning that graphene embedded in the developed nanostructure as an intelligent material can well highlight the variation of the effective refractive index of materials due to the defections and impurities available, which can manipulate the systems’ electric and vibrational properties [53,54].

**Figure 12:**
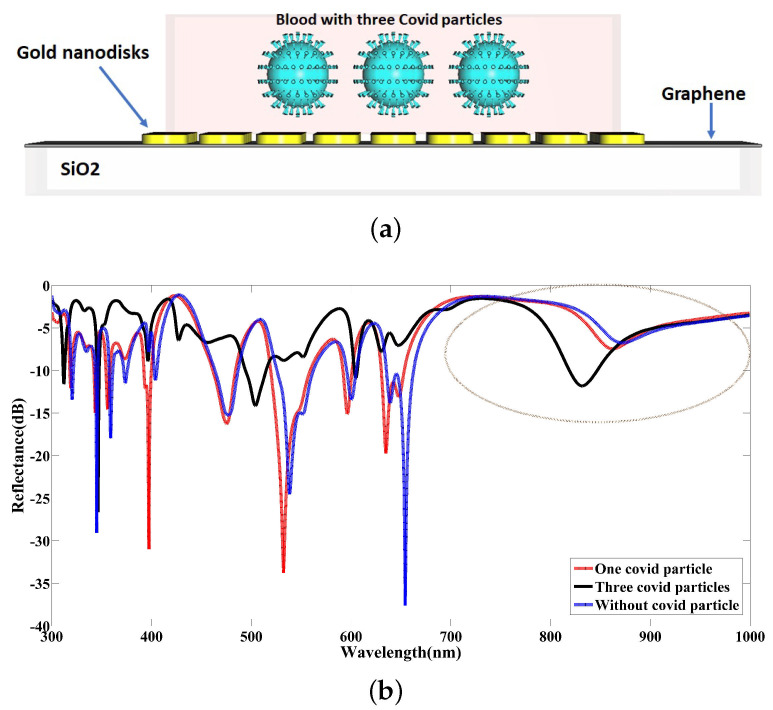
(**a**) A graphical screen of the proposed nanosensor under testing blood samples with a specific COVID-19 density; (**b**) the contrast of the reflected spectra for different blood samples with and without COVID-19 particles.

To explore the impact of the COVID-19 particle density on the reflectance parameter in the following, the number of COVID-19 models available inside the blood samples is increased to three particles of the same size, as illustrated in Figure 12a, and the spectral response is simulated in the reflection band. The distance of COVID-19 particles from each other is hypothesized to be 214 nm in the simulation model. The noteworthy point to make here is that a change in the (average) distance between the COVID-19 particles alters the effective refractive index of the medium because of the varying scattering profile of the electromagnetic waves, which means a change in the spectral response, as indicated in the previous section.

With a focus on the achieved results in Figure 12b and a comparison of them with the prior results, it can be understood that the developed nanosensor indicates a good functionality between 700 nm and 1000 nm, and it is able to detect well the further concentration of COVID-19 particles inside the blood sample under a regular variation. Moreover, the difference between the reflectance spectra in the visible range, such as 600– 700 nm, can be a wavelength band that the designed nanosensor shows a suitable performance for. As a result, existing COVID-19 particles in the standard blood sample change the effective refractive index of blood, and this variation under the various density of COVID-19 particles is different. For this reason, the reflectance spectra are deformed when the number of COVID-19 particles is increased, as illustrated in Figure 12b. As a remarkable point, it seems there is no trend over reflectance spectra arising from one particle blood sample and three particles blood samples. In detail, the interaction of the electromagnetic field components reflected from each COVID-19 particle leads to an interference effect in the resultant field that might be constructive or destructive. With the contrast of the results, it can also be understood that the role of spikes surrounding the COVID-19 particles causes this interference effect not to follow a specific trend when the number of COVID-19 particles changes from one to three, and we witness an irregularity in the spectral responses. Nevertheless, the main issue here is not how the spectra change with the number of COVID-19 particles, but instead that they are all different from the spectrum, corresponding to no COVID-19 particles present.

**Figure 13:**
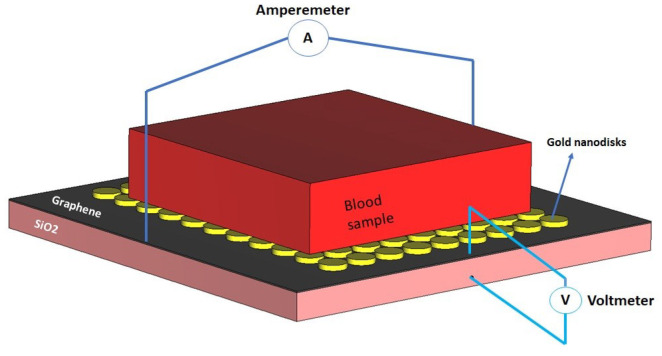
A graphical screen of the proposed nanosensor with electronic contacts, including voltmeter and amperemeter, attached to the nanodevice.

In attempting to simplify the measuring method of the designed kit and demonstrate the functionality of the nanosensor to gain a better perspective, we concentrate on the voltage variations between two layers, including graphene and silica, and the current variations available on the graphene sheet in order to explore the impact of the COVID-19 particles on the voltage and current parameters. It is worth mentioning that the incidence of the optical signal from the top of the structure manipulates the electron’s density upon the graphene layer embedded in the configuration of the nanosensor [55,56], which leads to current and voltage change. As such, it is expected that the presence of COVID-19 particles in the blood sample under test alters the concentration profile of electric charges, resulting in the variation of voltage and current. With this in mind, a current loop is defined in the implemented configuration in the CAD to obtain the current changes available on the graphene layer so that the primary point of the defined loop is placed on the center of the sided face of graphene, as can be seen in Figure 13. In addition, to achieve the voltage difference between two substrates, graphene and silica, we assume a curve branch to track voltage variations, as illustrated in Figure 13.

**Figure 14:**
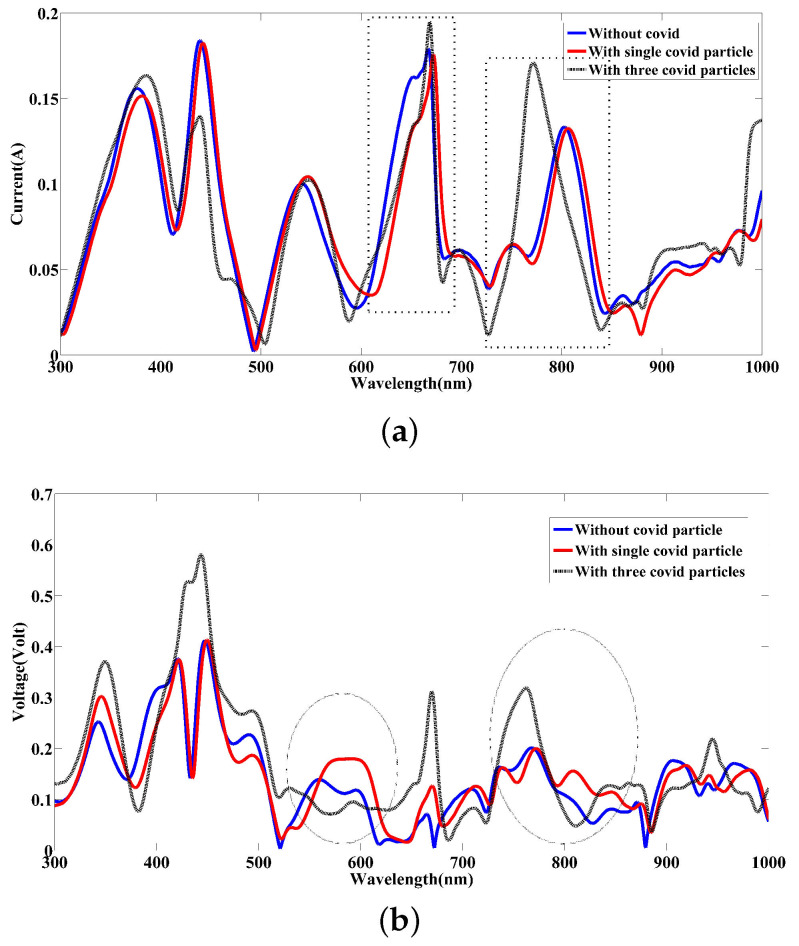
(**a**) The contrast of the current spectra resulted from the different blood samples with and without COVID-19 particles. (**b**) The contrast of the voltage spectra resulted from the various blood samples with and without COVID-19 particles.

Based on the simulated results presented in Figure 14a,b, as expected, the presence of the COVID-19 particle and especially its density can be well detected by the developed nanokit. With a focus on the calculated current curve over the desired range, the variations due to the presence of COVID-19 particles can be observed. For example, in NIR between 700 nm and 1000 nm, it can be seen that the peak of the current is over 0.15 A when the blood sample has a significant concentration of COVID-19 particles. In return, this amount is under 0.15 A for the blood cases with a single COVID-19 particle and without it. Further, the contrast of the current spectra related to the blood samples with and without a single COVID-19 particle reveals a shifting in the considered range associated with up and down, which can be well seen in the visible spectrum between 500 nm and 700 nm. Likewise, a similar result can also be concluded by viewing the voltage variation in Figure 14b, resulting from simulation. In detail, by observing the voltage spectra in the visible range and NIR, there is a considerable difference in voltage level obtained by the interaction between light, designed nanosensor, and blood samples. For instance, the voltage level achieved in the visible band ranging from 520 nm to 630 nm shows varying voltage levels between 0.08 V to 0.20 V for three blood samples used, which are clearly remarkable. By contrast, we are able to identify the healthy blood from the other blood samples. As a practical point, the level of current and voltage can also improve by means of stacking silica substrate and graphene sheet in a multilayer configuration [56].

From a practical standpoint, it is worth mentioning that adjusting the intensity of excitation light is necessary to avoid the electrolysis of the blood sample in the actual system. The simulation results indicate that the designed nanochip is able to detect the COVID-19 particles in the blood sample and well highlight the density of COVID-19 particles available in the blood samples, especially in the NIR band. Based on this optical simulation framework, it is well known that the performance of the proposed nanosensor can promote a new horizon for COVID-19 particle detection in sensory microelectronic graphene-based devices and enable us to gain an acceptable precision of the functionality of these kinds of electronic sensing chips and level up the reliability rate. As such, Optimizing the suggested nanosensor with different optical techniques may enhance the spectral responses in alignment with decreasing the error probability of COVID-19 identification. Further, the developed nanosensor has the potential to be realized as an antibody-labeled sensory kit by attaching a specific antibody to the gold nanodisks available on the graphene sheet to detect the COVID-19 particles in the blood samples. For instance, one could use a standard planar process from microelectronic fabrication consisting of optical lithography at a submicron wavelength and electron beam lithography at the nanoscale to fabricate the proposed optoelectronic sensor for COVID-19 particle detection [57].

## 4. Conclusions

In summary, this work presents a new modeling approach to optical characteristics of COVID-19 particles based on the established data in the literature for the first time. The results achieved in this study can be regarded as a preliminary achievement that makes the potential for fast detection of COVID-19 particles available in the blood sample through an accurate understanding of a coronavirus’s performance in the optical range. Our findings indicated that changes in the size of the COVID-19 particles, especially the spikes surrounding the core of COVID-19, result in common optical phenomena such as redshift and blueshift in the spectral response upon the reflection band. Furthermore, it is found that the reflectance spectra arising from light incidence on a concentration of COVID-19 particle models reveal a considerable reduction in the intensity of the reflected signal due to the high absorbance rate of the peripheral medium and the impact of the light incidence direction. In addition, the simulation results demonstrated that the distance between COVID-19 particles could influence the narrowing of the reflectance spectrum. Inspired by these simulations, we designed a graphene-based photonic structure associated with gold nanodisks as a nanosensor that has the potential to distinguish healthy blood samples from infected blood samples and make a fast detection mechanism. The developed nanosensor can be promoted as a promising candidate in the field of nanoelectronic kit design for sensory applications to identify abnormal blood samples with a simple test checklist through measuring current or voltage.

## Figures and Tables

**Table 1:** Variations of reflectance characteristic under the manipulation of spikes’ length.

Spike Length (nm)	Wavelength (nm)	Reflectance
h=10	793.73	0.9989
h=20	833.92	0.9723
h=30	873.01	0.9115
h=40	912.05	0.8424
h=45	927.86	0.7621

**Table 2:** Variations of reflectance characteristic due to the changing the radius of the COVID-19 model.

The Radius of the COVID-19 Model (nm)	Wavelength (nm)	Reflectance
r=30	672.78	1.0689
r=40	713.11	1.0639
r=50	753.25	1.0295
r=60	793.73	0.9996
r=70	833.92	0.9723

**Table 3:** Variations of reflectance characteristic due to the changing the base diameter of the spike proteins.

The Base Diameter of the Conical Spikes (nm)	Wavelength (nm)	Reflectance
D=6	837.18	0.9509
D=8	833.92	0.9663
D=10	829.07	0.9556
D=12	825.87	0.9924
D=14	821.12	0.9792

## Data Availability

Not applicable.
